# *Pedicularis resupinata* Extract Prevents Depressive-like Behavior in Repeated Corticosterone-Induced Depression in Mice: A Preliminary Study

**DOI:** 10.3390/molecules27113434

**Published:** 2022-05-26

**Authors:** Dong Wook Lim, Daeseok Han, Changho Lee

**Affiliations:** Division of Functional Food Research, Korea Food Research Institute, Wanju 55365, Korea; dwlim@kfri.re.kr

**Keywords:** *Pedicularis resupinata*, acteoside, corticosterone, HPA axis, depression, neurotoxicity, BDNF

## Abstract

Excessive corticosterone (CORT), resulting from a dysregulated hypothalamic–pituitary–adrenal (HPA) axis, is associated with cognitive impairment and behavioral changes, including depression. In Korean oriental medicine, *Pedicularis resupinata* is used for the treatment of inflammatory diseases such as rheumatoid arthritis. However, the antidepressant properties of *P. resupinata* have not been well characterized. Here, the antidepressant-like effects of *P. resupinata* extract (PRE) were evaluated in terms of CORT-induced depression using in vivo models. HPLC confirmed that acteoside, a phenylethanoid glycoside, was the main compound from PRE. Male ICR mice (8 weeks old) were injected with CORT (40 mg/kg, i.p.) and orally administered PRE daily (30, 100, and 300 mg/kg) for 21 consecutive days. Depressive-like behaviors were evaluated using the open-field test, sucrose preference test, passive avoidance test, tail suspension test, and forced swim test. Treatment with a high dose of PRE significantly alleviated CORT-induced, depressive-like behaviors in mice. Additionally, repeated CORT injection markedly reduced brain-derived neurotrophic factor levels, whereas total glucocorticoid receptor (GR) and GR phosphorylation at serine 211 were significantly increased in the mice hippocampus but improved by PRE treatment. Thus, our findings suggest that PRE has potential antidepressant-like effects in CORT-induced, depressive-like behavior in mice.

## 1. Introduction

Depression is one of the most serious global health concerns, with over 300 million individuals affected worldwide [1]. The recent COVID-19 pandemic has significantly induced mental disorders such as delirium, depression, anxiety, and insomnia, due to fear of disease, and the effects of social isolation [2,3]. Currently available first-line antidepressants are not completely effective and can cause a variety of undesirable side effects [4]. For these reasons, it is urgent to search for alternative antidepressants, particularly those involving the use of natural products [5]. Alternative approaches, including herbs such as *Hypericum perforatum* (i.e., St. John’s wort) [6] or increased intake of antioxidants with fruits and vegetables, have attracted interest as potential antidepressants [7]. According to the American Psychological Association (APA)’s guidelines for the treatment of depression, no difference in effects between St. John’s wort and second-generation antidepressants in some patients with depression [8]. Thus, it is necessary to develop new antidepressant agents with excellent efficacy and few side effects from natural products.

Excessive release of glucocorticoids, cortisol in humans, and corticosterone (CORT) in rodents led to dysregulation of the hypothalamic–pituitary–adrenal (HPA) axis, which is one of the most prominent neurobiological findings in the pathogenesis of depression [9]. Thus, increased HPA axis activity, glucocorticoid resistance, and increased plasma levels of cortisol are commonly observed in depression. It was reported that about 40–60% of depressed patients experience HPA axis disorders [10]. In addition, high levels of CORT have been demonstrated to induce depressive-like behaviors in animal models, as indicated by decreased sucrose consumption and significantly increased immobility time in the forced swim and tail suspension tests [11,12]. These findings suggest that a CORT-induced depression model is valid for evaluating the efficacy of potential antidepressants.

*Pedicularis resupinata*, an edible Korean wild plant belonging to the family Scrophulariaceae, has been used as an anti-inflammatory folk remedy in traditional Korean medicine. It is also distributed in eastern Asia, Japan, China, Mongolia, Siberia, Sakhalin, and Kamchatka [13]. Antioxidant effects of *P. resupinata* have been mainly reported in Korea [14]; however, not much is known about the efficacy of *P. resupinata*. Although little is known about the pharmacological efficacy of *P. resupinata*, the extract of the species of Pedicularis, which contains phenylpropanoid glycoside and acteoside as the main components, is known for its antioxidant effects [15]. Several studies have reported that acteoside has antioxidant [16], anti-inflammatory [17], neuroprotective [18], and hepatoprotective [19] properties. However, the antidepressant-like effects of *P. resupinata* extract on depressive mood have not yet been defined. In order to explore natural extracts with antidepressant efficacy, we conducted an in vitro assay to evaluate the neuroprotective effects of several Korean wild plants, among which *P. resupinata* extract showed a high neuroprotective potential, and it was determined that acteoside was the main component of *P. resupinata* extract.

In this study, we aimed to investigate the antidepressant-like effects of *P. resupinata* extract (PRE) in an animal model of depression established by repeated chronic administration of corticosterone (CORT) [20]. Depressive-like behaviors were evaluated using the open-field test (OFT), sucrose preference test (SPT), tail suspension test (TST), and forced swim test (FST). Furthermore, hippocampal glucocorticoid receptor and brain-derived neurotrophic factor expressions were also determined. 

## 2. Results

### 2.1. Effects of PRE on OFT in CORT-Injected Mice

**Figure 1:**
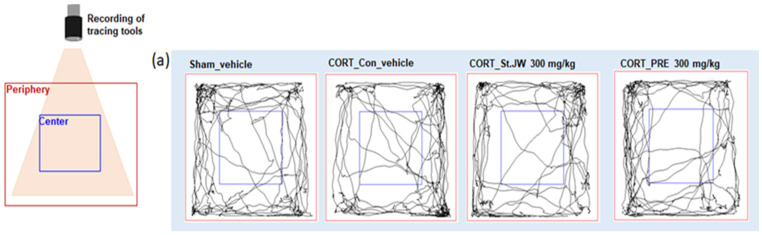

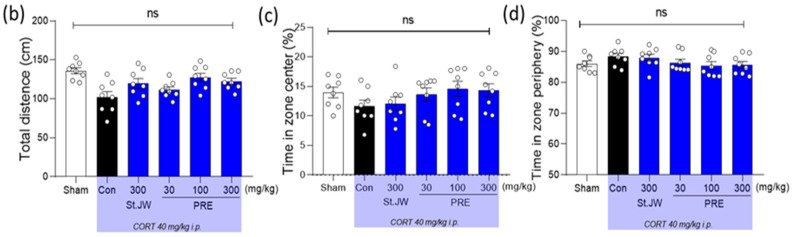
Effect of PRE on open filed test in CORT-induced depressive mice. A representative trace of locomotor activity across 5 min (**a**) in the OFT. There was no significant difference in total distance moved (**b**), time spent in the center (**c**), or periphery (**d**) of the OFT, between the treated groups. Results are presented as mean ± SEM (*n* = 8, per group). Differences among experimental groups were determined by analysis of variance (ANOVA) test. Con, control; CORT, corticosterone; St. JW, St. John’s wort extract; PRE, *P. resupinata* extract.

First, we examined the effect of PRE on CORT-induced alterations in the open-field test (OFT). As shown in Figure 1, CORT-injected mice did not show a significant change in locomotor activity (Figure 1a). In addition, there was no significant difference in the total distance moved (Figure 1b), time spent in the center (Figure 1c), or periphery (Figure 1d) of the OFT, between the PRE-treated groups. Their results indicated that administration of PRE is not related to an increase in motor activity in CORT-injected mice.

### 2.2. Effect of PRE on SPT in CORT-Injected Mice

**Figure 2:**
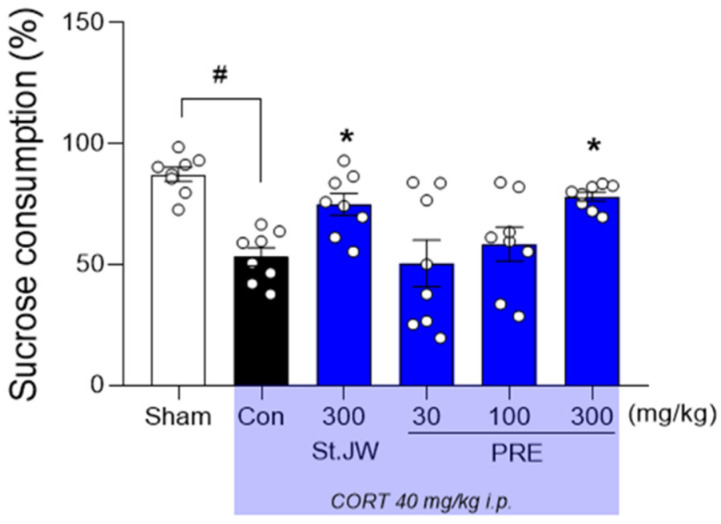
Effect of PRE on sucrose consumption in CORT-induced depressive mice. CORT injections significantly decreased sucrose consumption, while treatment with PRE at doses of 300 mg/kg significantly increased it. Results are presented as mean ± SEM (*n* = 8, per group). # *p* < 0.05 versus the sham group; * *p* < 0.05 versus the CORT-injected Con group. Con, control; CORT, corticosterone; St. JW, St. John’s wort extract; PRE, *P. resupinata* extract.

The repeated CORT injection significantly decreased the percentage of sucrose consumption, compared with the sham group (*p* < 0.05). However, it was shown that the reduced percentage of sucrose consumption was significantly restored in the group treated with St. John’s wort extract (*p* < 0.05). Similarly, PRE at a dose of 300 mg/kg significantly increased the percentage of sucrose consumption (*p* < 0.05) (Figure 2).

### 2.3. Effect of PRE on PAT in CORT-Injected Mice

**Figure 3:**
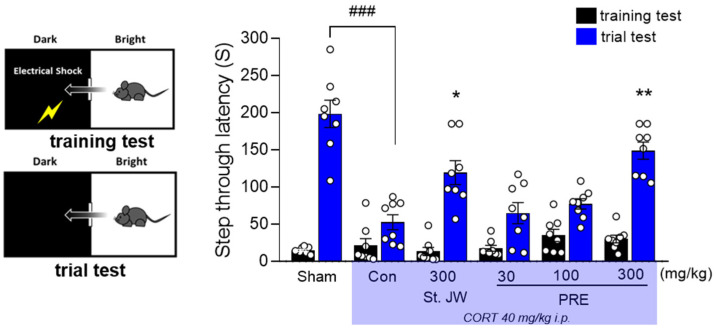
Effect of PRE on passive avoidance test in CORT-induced depressive mice. CORT injections significantly decreased step-through latency time (s), while administration of PRE at doses of 300 mg/kg significantly increased latency time. Results are presented as mean ± SEM (*n* = 8, per group). ### *p* < 0.001 versus the sham group; * *p* < 0.05 and ** *p* < 0.01 versus the CORT-injected Con group. Con, control; CORT, corticosterone; St. JW, St. John’s wort extract; PRE, *P. resupinata* extract.

Memory impairment is commonly seen in many stress-related disorders, including depression [21]. Thus, we examined the effect of PRE on memory impairment caused by CORT injection in mice. As shown in Figure 3, CORT-injected mice exhibited memory impairment, as the step-through latency time (s) was significantly decreased, compared with the sham group (*p* < 0.001). However, administration of PRE at doses of 100 and 300 mg/kg significantly improved the step-through latency time (s). These effects were similar to those observed for St. John’s wort (*p* < 0.05).

### 2.4. Effect of PRE on TST and FST in CORT-Injected Mice

**Figure 4:**
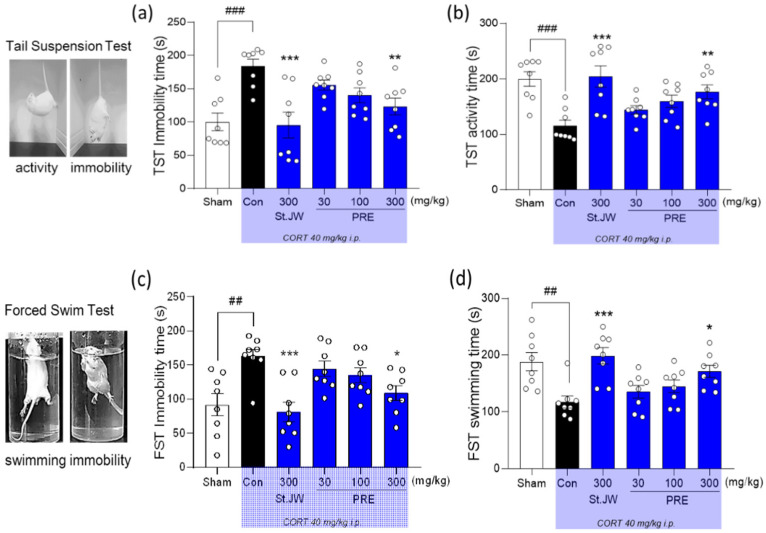
Effect of PRE on tail suspension test and forced swim test in CORT-induced depressive mice. CORT-injected mice had significantly increased immobility and decreased activity, including swimming, while mice treated with PRE at doses of 300 mg/kg showed significant improvements, with a decreased immobility (**a**,**c**) times (s) and increased activity (**b**) and swimming (**d**) times (s). Results are presented as mean ± SEM (*n* = 8, per group). ## *p* < 0.01 and ### *p* < 0.001 versus the sham group; * *p* < 0.05, ** *p* < 0.01, and *** *p* < 0.001 versus the CORT-injected Con group. Con, control; CORT, corticosterone; St. JW, St. John’s wort extract; PRE, *P. resupinata* extract.

Repeated CORT injections had a significant effect on mice behavior in the tail suspension test (TST) and forced swim test (FST) [22]. The CORT-treated control group exhibited depression-like behavior characterized by significantly increased immobility time in TST and FST. An increase in immobility time is interpreted as depression-like behavior in animal models of depression [23]. As expected, the group that was administered St. John’s wort extract at a dose of 300 mg/kg showed a significant decrease in immobility time, compared with the control group, which was similar to previously published preclinical studies [24]. Similar efficacy was exhibited by the administration of PRE at a dose of 300 mg/kg in the TST and FST (Figure 4). These results indicate that administration of PRE had antidepressant-like effects in a CORT-induced depression model.

### 2.5. Effect of PRE on GR and BDNF Expression

**Figure 5:**
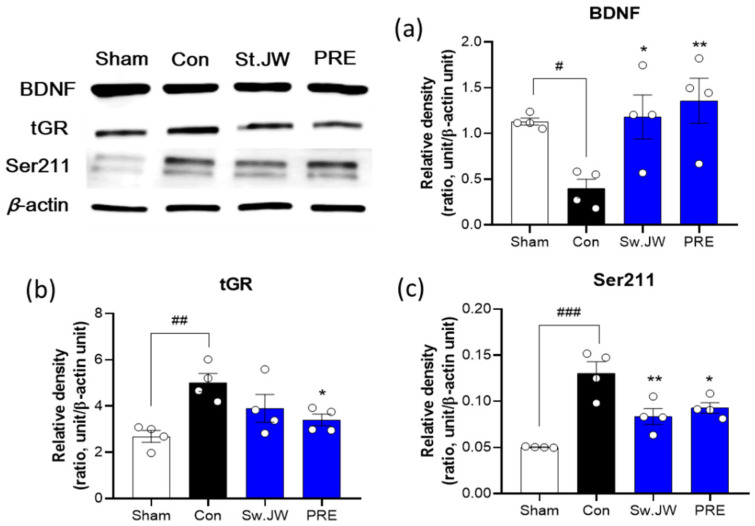
Effect of PRE on glucocorticoid receptor activity in CORT-induced depressive mice. Hippocampal Western blot analysis revealed that CORT injections significantly decreased BDNF (**a**) expression and increased tGR (**b**) and Ser211 (**c**) expression in the hippocampus, while treatment with PRE at 300 mg/kg significantly attenuated the effect. Results are presented as mean ± SEM (*n* = 4, per group). # *p* < 0.05, ## *p* < 0.01, and ### *p* < 0.001 versus the sham group; * *p* < 0.05, and ** *p* < 0.01 versus the CORT-injected Con group. Con, control; CORT, corticosterone; St. JW, St. John’s wort extract; PRE, *P. resupinata* extract.

After FST, the hippocampal tissue from each mouse was isolated from the whole brain for Western blot analysis. Depression has been correlated with alternations in glucocorticoid receptor (GR) signaling [25]. Prolonged or excessive exposure to CORT leads to neuronal damage, particularly in the hippocampus, which is enriched with GR [26]. GR can be phosphorylated at serine 211 (Ser211) and enhanced Ser211 had a stronger correlation with depression [27]. Indeed, chronic glucocorticoid stress, which stimulates GR activation, causes downregulation of the expression of brain-derived neurotrophic factor (BDNF) [28]. As shown in Figure 5, the hippocampal BDNF levels were significantly decreased, whereas total GR (tGR) and GR phosphorylation at Ser211 were significantly increased in the CORT-treated control group. However, PRE at a dose of 300 mg/kg significantly increased BDNF levels and decreased tGR and Ser211 levels within the hippocampus. These results indicate that administration of PRE improved the alternation of the GR expression and increased the activity of BDNF, thereby alleviating depressive behavior, including memory impairment.

## 3. Discussion

In the present study, we determined that PRE administration was sufficient to induce antidepressant-like effects in a well-established CORT-induced mouse model of depression [20]. We found that PRE administration facilitated antidepressant-like behavior and significantly reduced immobility time in TST or FST, without affecting locomotor activity. Moreover, PRE attenuated the CORT-induced overactivation of GRs and improved cognitive function by increasing BDNF levels. These neuroprotective effects were also confirmed in human neuroblastoma SH-SY5Y cells. These results suggest that PRE could ameliorate stress-hormone-induced depressive behaviors by modulating GR-mediated BDNF expression in mice.

HPA axis dysfunction is a well-known risk factor for depression, including cognitive impairment [29]. The fact that patients with depression exhibit cortisol hypersecretion due to disturbance of the glucocorticoid negative feedback system [30] suggests an association between HPA axis dysfunction and depression [31]. It has also been reported that exposure to high levels of CORT causes damage to the brain, particularly the hippocampus, one of the brain regions where GRs are highly concentrated [32], leading to symptoms such as cognitive decline or depression. Patients with depression have been reported to have smaller hippocampal volume, compared with healthy controls [33]. Similarly, in animal studies, chronic stress or repeated injections of high-dose CORT induce HPA axis dysfunction, resulting in depression-like behavior, which is modulated by antidepressant treatment [34,35]. Immobility in behavioral tasks related to animal models of depression has been coined as behavioral despair or learned helplessness [36]. Increased immobility in TST or FST is well-known to parallel human depression-related symptomology, and this state of immobility is significantly improved when treated with antidepressants [37]. We determined that immobility time was significantly increased in TST and FST in mice receiving 40 mg/kg of CORT daily, and this study showed a similar trend to that of a previous study [38]. As expected, we found that the PRE-treated group had significantly reduced immobility time in TST and FST, without any changes in locomotor activity, especially at the 300 mg/kg dose; this antidepressant-like effect was similar to that of the group receiving St. John’s wort extract as a positive control. 

GR is a phosphoprotein that becomes hyper-phosphorylated upon binding to glucocorticoids [39]. Excessive GR activation by high concentrations of CORT impairs hippocampal neurogenesis, suggesting that normalization of GR function is critical to the antidepressant effect [40]. It has been reported that the expression of hippocampal GR is significantly upregulated by exposure to chronic mild stress-induced depression in animal models [41]. GR activation is critically dependent on the phosphorylation status of specific serine residues such as Ser211 [42]. It has been reported that phosphorylation at Ser211 facilitates nuclear translocation and increases GR transcriptional activity [43]. Our results also showed that total GR protein levels were increased by chronic CORT exposure, suggesting that prolonged exposure to CORT resulted in the upregulation of GR expression and increased GR phosphorylation at Ser211, whereas PRE normalized the CORT-induced increase in GR protein and GR phosphorylation at Ser211. BDNF, a member of the neurotrophin family, is highly expressed in the hippocampus and is mainly involved in cognitive function and mood changes [44]. Clinical studies have demonstrated that BDNF is an important factor in the pathogenesis of depression [45]. It has been reported that low serum BDNF levels in patients with depression, were significantly increased by antidepressant treatment [46]. We determined that the level of hippocampal BDNF was significantly decreased in the CORT-treated control group, which was correlated with cognitive decline in the passive avoidance test (PAT), following CORT injection in mice. As expected, we found that PRE prevented the CORT-induced reduction in BDNF levels in the hippocampus, and PRE treatment promoted cognitive improvement, as evidenced by increasing step-through latency time in the PAT.

Plants of the genus *Pedicularis* are rich sources of natural compounds, fatty acids, alkaloids, steroids, lignans, neo-lignans, tannins, ionones, phenylpropanoid glycosides, phenylethanoid glycosides, flavonoids, xanthones, iridoids, secoiridoids, phenyl-glycosides, organic acids, polyols, saccharides, and amino acids [47]. *P. resupinata* has been identified for their phytochemical compounds, alaschanioside A, alaschanioside C, syringaresinol-4-O-β-d-glucoside, verbascoside, 2,3-O-diacetyl-martynoside, leucosceptoside A, plantarenaloside, euphroside, boschnaloside, gardoside methyl ester, and geniposidic acid [47,48]. In particular, phenylpropanoid glycosides are a class of natural substances of plant origin with interesting biological activities and pharmacological properties [49]. There have been studies on the biological activities of phenylpropanoid glycoside, which are potent antioxidants [50] and free radical scavengers [51]. It has been reported that oxidation-derived reactive oxygen species increase monoamine oxidase in neurons and astrocytes, leading to deficits in memory function and depression [52]. The antioxidant effect is the major function of acteoside, one such phenylpropanoid glycoside, and has widely been demonstrated [53]. Serval studies showed that acteoside might protect C57 mice against MPTP-induced neuronal damage [54] and suggested that the neuroprotective effect of acteoside might be related to its function of reducing ROS levels. Our HPLC analysis confirmed that acteoside was the main compound from PRE. Although we did not perform HPLC-based activity profiling of compounds from PRE, with our HPLC analysis, at least, we could state that the antidepressant-like effects of PRE might be associated with high contents of the acteoside. A limitation of this study is that our in vivo findings present a portion of the animal’s behavior results, and the dose does not reveal the bioavailability of the active compounds of PRE. Thus, the efficacy of active PRE compounds needs to be tested in vivo. Moreover, further studies are necessary to determine whether active compounds from PRE act on the central nervous system through blood–brain barrier penetration. Taken together, our results indicate that PRE upregulates BDNF levels against CORT-induced depression-like phenotypes by regulating the HPA axis through normalizing GR function via inhibition of Ser211-mediated GR phosphorylation.

## 4. Materials and Methods

### 4.1. Sample Preparation

**Figure 6:**
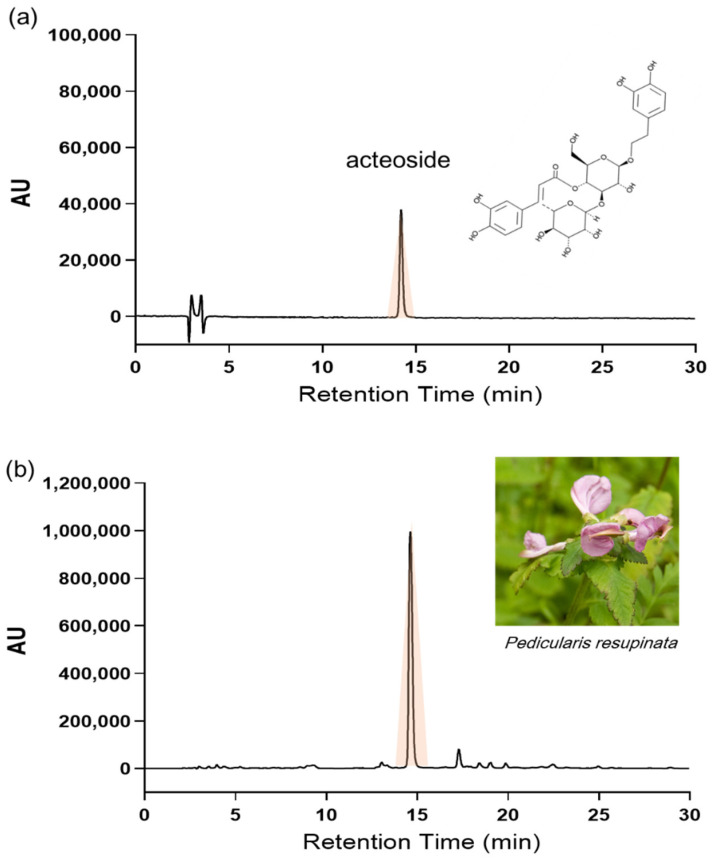
HPLC chromatogram of (**a**) acteoside as a standard compound and (**b**) Pedicularis resupinata extract. The concentration of acteoside was 0.78 ± 1.15 mg/g PRE.

MeOH extract of *Pedicularis resupinata* (PRE, voucher specimen No. KPM032-051) was obtained from the Korea Plant Extract Bank of the Korea Research Institute of Bioscience and Biotechnology (Daejeon, South Korea). The plant was collected from Jeollabuk-do, Korea, in 2007. The dried plant (100 g) was added to 1 L of methyl alcohol 99.9% (HPLC grade) and extracted at room temperature by using an ultrasonic extractor (SDN-900H, SD-ULTRASONIC, Seoul, Korea). After filtration and drying under reduced pressure, the methanol extract (PRE, 3.09 g) was obtained. PRE was dissolved in 50% HPLC-grade MeOH and analyzed by HPLC (Jasco, Tokyo, Japan) using a Waters Symmetry ^®^ C18 column (4.6 × 250 mm, 5 μm). The mobile phase was established using 0.2% formic acid (A) and ACN/MeOH (60:40, *v*/*v*) (B), via gradient elution. The chromatographic separation was processed with a solvent (A) gradient that decreased from 80% to 50% within 40 min of running time and then increased to 80% with a 45 min running time. The injection volume of PRE was 10 μL with a flow rate of 1 mL/min and was detected at 280 nm using a PDA detector. The concentration of acteoside was 0.78 ± 1.15 (mean ± SD) mg/g. Representative chromatograms of acteoside and PRE are shown in Figure 6.

### 4.2. Animals and Treatments

**Figure 7:**
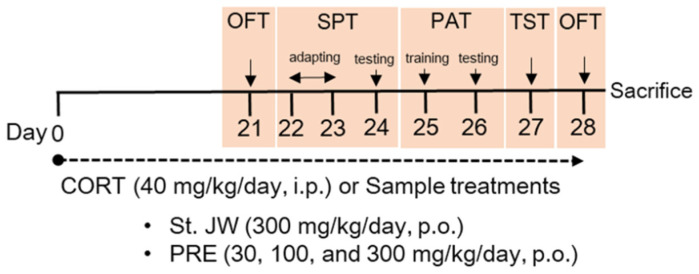
Experimental design for animal model of CORT-induced depression-like behavior. CORT, corticosterone; OFT, open-field test; SPT, sucrose preference test; PAT, passive avoidance test; TST, tail suspension test; FST, forced swim test; St. JW, St. John’s wort extract; PRE, *P. resupinata* extract.

ICR mice (male, 7 weeks old, weighing 25–28 g) were purchased from KOATECH Animal Inc. (Pyeongteak, Korea). The mice had ad libitum access to food and water in a climate-controlled chamber (temperature, 21 ± 2 °C and light–dark cycle, 24 h, lights on at 07:00, and lights off at 19:00). All mice (*n* = 4 per cage) were habituated to the facility for 1 week prior to starting the experiments. All animal experiments were approved by the Institutional Animal Care and Use Committee of the Korea Food Research Institute (KFRI-M-19016). To induce depression-like behavior, a high dose of CORT was intraperitoneally (i.p.) injected according to a previous study. CORT (Sigma-Aldrich, St. Louis, MO, USA) was dissolved in 0.9% (*w*/*v*) saline containing 0.1% dimethyl sulfoxide (DMSO; Sigma-Aldrich) and 1% Tween-80. St. John’s wort (80% MeOH extract, dried powder of Hypericum japonicum), which was the positive control, and PRE was dissolved in distilled water. The mice were randomly assigned to six groups (*n* = 8): (1) sham, (2) control, (3) St. John’s wort 300 mg/kg, (4) PRE 30 mg/kg, (5) PRE 100 mg/kg, and (6) PRE 300 mg/kg. Mice in the control and sample-treated groups were orally administered CORT (40 mg/kg, i.p.) once daily, while those in the sham group were treated with an equal volume of vehicle. After 3 weeks of treatment, the mice were tested in depression-related behavioral tasks, beginning 1 h after sample administration, as per the experimental scheme (Figure 7), and were then sacrificed for Western blot analysis.

### 4.3. Open-Field Test (OFT)

The mice were placed in the center of an open field maze (50 × 50 × 50 cm), and their movements were recorded using a video camera; behaviors, total distance, and time in the center and periphery zones were tracked for 5 min using the SMART video tracking system (SMART v3.0, Panlab SL, Barcelona, Spain).

### 4.4. Sucrose Preference Test (SPT)

SPT was performed 24 h after OFT, following a previously established protocol. Two bottles containing the 1% sucrose solutions were placed in a cage for 24 h. Next, one of the bottles of the sucrose solution was placed with water for 24 h. The mice then were placed in cages individually with free access to the two bottles for 24 h, and the consumed volumes were recorded. Sucrose preference was calculated as follows: consumption (%) = [sucrose intake/(sucrose intake + water intake)] × 100.

### 4.5. Passive Avoidance Test (PAT)

PAT was performed using the passive avoidance apparatus (GEMINI, SD instruments, San Diego, CA, USA), as previously described. The PAT experiment was conducted across 2 days. In the training trial, each mouse was placed in a safe zone with a closed door. After acclimatization for 1 min, the door opened automatically, and the mice were allowed to enter the dark zone. When the mice entered the dark zone, an electrical foot shock of 0.5 mA for 3 s, was elicited. On the next day, the mice were again placed in the safe zone, and the latency to enter the dark zone was recorded.

### 4.6. Tail Suspension Test (TST) and Forced Swim Test (FST)

In the case of TST, each mouse was suspended by its tail using adhesive tape and attached to a hook that automatically measured its movement. The immobility time during the 6 min test was measured using an automated TST apparatus (BioSeb, Chaville, France). In the FST, the mice were forced to swim for 6 min in a transparent Plexiglas cylinder (height, 13 cm; diameter, 24 cm) filled with water to a depth of 10 cm (temperature, 22–24 °C). The immobility time during the last 4 min was measured using a SMART video tracking system (SMART v3.0, Panlab SL, Barcelona, Spain).

### 4.7. Western Blotting 

Hippocampal brain tissues were homogenized in RIPA buffer containing a protease and phosphatase inhibitor cocktail. The quantified proteins (20 μg) were separated by 10% sodium dodecyl sulfate–polyacrylamide gel electrophoresis and transferred onto polyvinylidene fluoride membranes (Millipore, Billerica, MA, USA). The membranes were blocked with 4% skim milk in Tris-buffered saline with 1% Tween-20 for 40 min, and subsequently probed with the following primary antibodies: BDNF mouse monoclonal antibody (1:1000 dilution, sc-65514, Santa Cruz Biotechnology, CA, USA), glucocorticoid receptor (1:1000 dilution, sc-393232, Santa Cruz Biotechnology, CA, USA; GR phosphorylation at serine 211 (Ser211, 1:1000 dilution, #4161, Cell Signaling, MA, USA), and anti-β-actin rabbit polyclonal antibody (1:1000 dilution, #4967, Cell Signaling, MA, USA) overnight at 4 °C in 3% skim milk in TBST. After incubation with the horseradish peroxidase–linked secondary antibody for 2 h, immunoreactive proteins were detected using a chemiluminescence detection system (LI-COR Biosciences, Lincoln, NE, USA) and then analyzed using ImageJ software (NIH, Bethesda, MD, USA).

### 4.8. Statistical Analyses

Statistical analyses were performed using Student’s *t*-test for two-sample comparisons and one-way analysis of variance, followed by Tukey’s post hoc test using Prism 8 (GraphPad Software, Inc., San Diego, CA, USA) for multigroup comparisons. All data are presented as the mean ± SEM. Differences were considered significant at *p* < 0.05. The sample size of 8 mice for each group was justified on the basis of a pilot experiment showing that the sample SD (s) for measurements of immobility time was about 5% of the measured value and the average immobility time expected difference (d) between vehicle-treated mice and PRE-treated mice was about 50 s. Assuming that α = 0.05 and 1 − β = 0.9, the formula used was *n* (sample size) = 1 + 21 × (s/d)^2^ [55]. The formula gave 7.89, which was increased to 8 in case of unexpected experimental problems.

## 5. Conclusions

To our knowledge, this is the first study evaluating the antidepressant-like effects of PRE on CORT-induced depressive-like behaviors in mice. We found that PRE prevented CORT-induced depression-like behavior and improved cognitive function. These effects may be caused by the regulation of GR function via inhibition of Ser211-mediated GR phosphorylation and BDNF levels, which are involved in the inhibition of neuronal loss. Our results suggest the beneficial effects of PRE on the development of depressive behaviors in mice and, therefore, provide potential natural antidepressants.

## Data Availability

The data presented in this study are available on request from the corresponding authors.

## Sample Availability

Samples of the compounds are not available from the authors.
